# Assessing clinical outcomes of modified laparoscopic gastrostomy in children: a case control study

**DOI:** 10.1186/s12893-022-01515-0

**Published:** 2022-02-21

**Authors:** Hussein Naji, Aafia Gheewale, Ebtesam Safi, Faiz Tuma

**Affiliations:** 1 Mediclinic Parkview Hospital, Dubai, UAE; 2 Mohammed Bin Rashid University of Medicine and Health Sciences, Dubai, UAE; 3 Central Michigan University College of Medicine, Mount Pleasant, MI USA; 4 Western Michigan University Homer Stryker M.D. School of Medicine, Kalamazoo, MI USA

**Keywords:** Laparoscopic gastrostomy, Postoperative complications, U suture technique, Seldinger technique, Button, Children

## Abstract

**Background:**

With gastrostomy becoming a common surgical procedure within the pediatric population surgeons continued to introduce modifications on the procedure to overcome some of the challenges and minimize complications. Modified U-stitches laparoscopic gastrostomy is gaining favor in some centers including the center of this study. Hence, this study was conducted to evaluate and compare its outcomes.

**Methods:**

Eighty-nine gastrostomy procedures performed between 2013 and 2020 were reviewed to evaluate the surgical outcomes of a novel modified U-stitches laparoscopic gastrostomy (MLG) to the standard laparoscopic gastrostomy (LG) in children. The main outcome measured is the rate of postoperative complications encompassing dislodgement of gastrostomy button, leak around button, local infection, and development of granulation tissue post-surgery which is compared between the two population groups.

**Results:**

The rate of leak around the button was found to be significantly less in the MLG (4%) compared to (15%) in the traditional LG approach with a p-value of 0.03. However, the overall complication rate for MGL is 63%; while it is 73% for LG.

**Conclusions:**

The modified U-stitches laparoscopic gastrostomy has a lower rate of complications in comparison to the standard laparoscopic gastrostomy making it a preferred technique for gastrostomy placement in children.

## Background

According to the European Society for Clinical Nutrition and Metabolism (ESPEN), gastrostomy tube is indicated in patients who require enteral feeding for more than 2 weeks [1]. The goal of this feeding supplementation is to prevent significant weight loss, nutritional deficiencies, assist with growth in children and improve their quality of life.

With the advancement of minimal invasive surgeries, newer modifications and techniques of feeding gastrostomy have been developed including percutaneous endoscopic gastrostomy, percutaneous radiologic gastrostomy and laparoscopic gastrostomies [2]. While all methods have varying complications, surgeons are continuously trying to evaluate and choose the appropriate method to improve patient outcome and minimize complications [3–5].

Percutaneous endoscopic gastrostomy (PEG) has gained popularity among surgeons after it was first introduced in the 1980s [6, 7]. With the continuous advances in laparoscopy, laparoscopic gastrostomy (LG) became more available afterward [5]. It is a minimally invasive procedure where the gastrostomy button can be placed directly (instead of a feeding tube), with shorter hospital stay, quicker recovery and fewer complications [2, 3, 5, 8]. In addition, the risk of accidental gastro-enteric fistula development was found to occur in lower rates in LG compared to PEG [9, 10].

The standard laparoscopy technique allows for easy placement of the gastrostomy with direct visualization and manipulation of the stomach yielding a minimized risk of unintentional visceral injury [11]. A 5-mm umbilical port introduced to the peritoneal cavity. The surgeon explores the abdominal cavity with a 5-mm (30°) telescope through that umbilical port. A 5-mm stab incision made over the designated site for the gastrostomy tube placement. The gastric wall is then grasped with a 5-mm laparoscopic Babcock forceps and brought to the wound site. Once exteriorized, the gastric wall is fixed to the anterior abdominal fascia with four sutures. The incision is sometimes enlarged up to an additional 1cm for placement of the sutures. A gastrotomy is created at the center of the sutures by diathermy. A balloon gastrostomy button is then inserted over a probe.

**Fig. 1:**
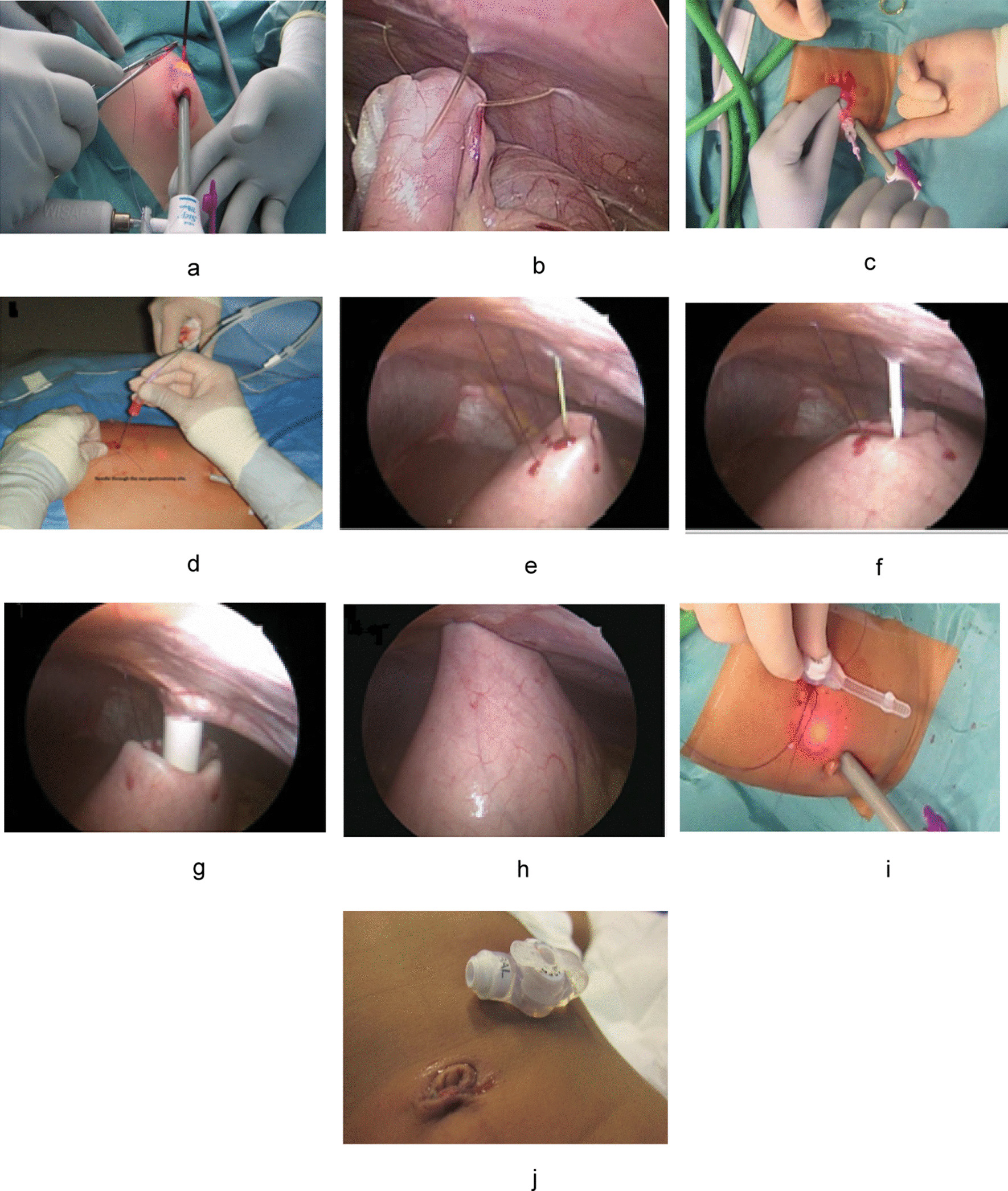
Technique of MLG

While all methods have varying complication rates, surgeons try constantly to improve patient outcome. In line with these efforts of improvement, a novel technique was introduced in 2017 at our pediatric surgery department with a modified U-stitches laparoscopic gastrostomy. This modification consists of a combination of hidden U-stitches placed under direct vision of laparoscopy to anchor the stomach to the abdominal wall and using Seldinger technique to insert the gastrostomy button (Appendix 1, Figure 1).

The aim of this study is to compare the rate of complications of two different laparoscopic techniques of a gastrostomy button placement in a pediatric population at a local institution.

## MLG operative technique

A 5-mm umbilical port introduced to the peritoneal cavity. A 3-mm laparoscopic grasper introduced through a stab incision in the upper left quadrant. The anterior wall of the stomach is grasped near the greater curvature away from the pylorus to avoid obstructing the gastric outlet. A 2:0 Vicryl (½ circle Taper Point Plus 44 mm Needle) is used to place an anchoring U-stitch through the abdominal wall and then through the full thickness of the gastric wall then the abdominal wall under direct vision with laparoscopic guidance. A second U-stich is placed in the same fashion but lateral to the position of the grasper. The grasper is then removed while the stomach is anchored by the 2 U stitches. A needle (18-G) is inserted between the U stitches through the same opening on the abdominal wall that was used for the grasper and pushed under direct laparoscopic vision through the gastric wall to the stomach (Fig. 1A, B). A guidewire is then introduced through the puncture needle to the stomach. The needle is removed leaving the guidewire in place (Fig. 1C, E). A telescoping dilator with a built-in peel-way external sheath is slowly introduced over the guidewire in a step-by-step telescoping manner (Fig. 1F, H). After sufficient dilation, the dilator is removed and an over-the-wire stoma measuring device is inserted to determine the appropriate length of the gastrostomy button to be placed. Next, the dilator is reinserted, and the peel-away external sheath is introduced into the gastric lumen. The dilator is then removed, leaving only the external sheath with the guidewire in place. A suitable size balloon gastrostomy button with an appropriate length shaft is inserted into the external sheath over the guidewire and the external sheath is slowly peeled off. The guidewire is removed to leave only the gastrostomy button in place (Fig. 1I, J). Each U stitch is then tied subcutaneously after pulling its 2 arms by a Mosquito artery forceps introduced in the subcutaneous space. The 2 U stitches will ensure fixating the stomach to the abdominal wall while the button’s balloon will secure the button in its place and prevent its dislodgment.

## Methods

After obtaining approval from the Mediclinic Middle East Research and Ethics Committee (MCME.CR 161. MPAR. 2020) and Dubai Scientific Research Ethics Committee (DSREC), DHA (DSREC-01/2021_19), all children who underwent laparoscopic gastrostomy button placement between 2013 and 2020 were considered. Data was collected from the electronic medical records without personal identifiers. Patients were divided into two groups according to the type of surgical procedure. The first group (control group) includes 41 patients who underwent the standard laparoscopic gastrostomy button placement (LG) between 2013 and 2016. While the second group includes 48 patients who underwent the newly introduced modified laparoscopic gastrostomy (MLG) between 2017 and 2020.

Children who underwent open surgical or PEG tube placement, children who underwent a concomitant procedure such fundoplication, or children who were not available for follow up were excluded from the study.

The following variables were collected: demographic data (age, sex and weight of patients), indications for gastrostomy, comorbidities, operative time and post-operative complications including leakage, infection, granuloma formation, dislodgment of the button, and the follow up progress for a period of 1 year (at 1, 6 and 12 months).

## Results

**Fig. 2:**
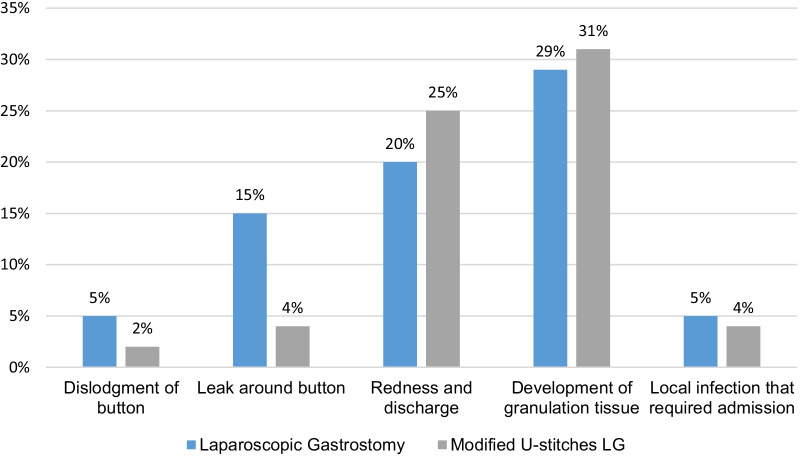
Bar graph representing the rate of complications

A total number of 89 children were enrolled in the study. The median age was 1.5 years (ranged between 1 month to 17 years), with no major intraoperative complications. There were no procedure related deaths nor was there any necessity to switch to open surgery. The median operating time was 55 min for the LG and 56 min for the MLG as shown in Table 1. The postoperative complications are summarized in Table 2; with the most common complication for both procedures being formation of granulation tissue as seen in 15 patients (31%) in group one versus 12 patients (29%) in group two. Leakage around the button was markedly less in the second group (4% versus 15 %) as can be seen in Fig. 2.

The overall rate of complications however was still less with the MLG group. This is proven with the p-value 0.03 (< 0.5) resulting in a significant difference. While the study stands internally valid to its sample population, it also is externally valid with the evidence of a low standard error (0.07), denoting a close relationship between the sample and wider population.

## Discussion

As technology enhances more advancement of surgical procedures and as our understanding and close follow up of patients improves, surgeons will keep introducing new modifications on their techniques and approaches. Gastrostomy tube placement is not different from this consideration. Since the introduction of the percutaneous gastrostomy feeding tube, several techniques made their way to implementation. With each technique or approach, healthcare providers soon identify issues when they appear and start searching for solutions and improvement. However, there are issues that are inherited to the type of the procedure or the disease that are difficult to eradicate such as some of the complications of gastrostomy feeding tube placement. Furthermore, expertise might be variable among surgeons. Hence, it is prudent to study and identify the appropriate approach for the right procedure and patient population within the available set of surgical skills. In our case, the MLG was sought to be more appropriate and can provide better patient outcomes but needed objective evaluation of the results.

Laparoscopic gastrostomy has many overall advantages for children who require long-term enteral nutrition [9]. The surgeon has a better visual field thereby lowering the risk of perforation of hollow viscous and vascular injury. Laparoscopic visualization prevents accidental gastro-enteric fistula formation which is encountered in 1.27% of PEG [9]. Placement of a button directly is advantageous to the patient and is much easier to handle than tube gastrostomy. With the newer approach of MLG, the rate of postoperative complications has decreased, particularly leakage around the button and dislodgment of the button making this modified technique an enhanced surgical option for gastrostomy placement. This improvement in complication rate is consistent with the recent studies [5, 12].

The reason of this lower complication rate is likely due to the placement of the U stitches which fixate the stomach to the abdominal wall in an efficient way with minimal handling of the gastric tissue as the stomach remained inside the abdominal cavity at all times with no exteriorization. The stitches are buried under the subcutaneous tissues giving wonderful cosmetic results; particularly with the use of a 3 mm incision. The modification of burying the U stitches underneath the skin adds another benefit by avoiding the need for removal of those stitches. This addition differentiates our technique from that described by Aprahamian [13]. The minimal intervention needed to introduce the dilators in the Seldinger technique further enhances the cosmetic aspect with minimal damage to the tissues. We believe that this minimal handling of tissues and the small incision are the reason for the reduced leakage rate around the button.

This modified technique of gastrostomy is simple, effective and takes approximately the same operative time as the standard surgery. Improving quality of life after surgeries is an ongoing goal for surgeons; hence placement of the button directly with minimal complication rate and providing a good cosmetic result is giving an advantage of MLG over the standard LG.

## Conclusions

MLG is a less invasive surgical procedure compared to the standard LG with a decrease in complication rates. Further studies are needed to verify the underlying reasons of improved outcomes.

## Data Availability

The datasets used and/or analyzed during the current study are available from the corresponding author on reasonable request.

## Appendix 1

See Figs. 1 and 2.

## Appendix 2

See Tables 1 and 2.

**Table 1:** Age of patients and the median operating time

	Laparoscopic gastrostomy (LG)	Modified U-stitches LG
No. of patients	41	48
Median age	1.5 years (range 1 month–17 years)	1.5 years (range 1 month–14 years)
Median operating time	55 min (range 46–94)	56 min (range 3–112)

Controls: patients undergoing the standard laparoscopic gastrostomy

Cases: patients undergoing the modified U stitch LG

**Table 2:** Summary of the postoperative complications of the two different procedures

Complications	Laparoscopic gastrostomy (LG)	Modified U-stitches LG
Number of patients	41	48
Dislodgement of button	2 (5%)	1 (2%)
Leak around button	6 (15%)	2 (4%)
Redness and discharge	8 (20%)	12 (25%)
Development of granulation tissue	12 (29%)	15 (31%)
Local infection that required admission	2 (5%)	2 (4%)

## Abbreviations

MLG: Modified U-stitches laparoscopic gastrostomy
LG: Standard laparoscopic gastrostomy
ESPEN: European Society for Clinical Nutrition and Metabolism
PEG: Percutaneous endoscopic gastrostomy
